# Field measurement of local wind environment on the approach deck of a suspension bridge in mountain terrain

**DOI:** 10.1038/s41598-022-19849-3

**Published:** 2022-09-19

**Authors:** Mingjin Zhang, Fanying Jiang, Jingyu Zhang, Jingxi Qin, Xulei Jiang, Yongle Li

**Affiliations:** 1 Department of Bridge Engineering, Southwest Jiaotong University, Chengdu, 610031 China; 2 Wind Engineering Key Laboratory of Sichuan Province, Southwest Jiaotong University, Chengdu, 610031 China; 3 China Railway Major Bridge Reconnaissance & Design Institute Co., Ltd., Wuhan, 430050 China; 4 Department of Civil and Environmental Engineering, University of California, Los Angeles, CA 90050 USA

**Keywords:** Civil engineering, Engineering

## Abstract

The local wind environment above the bridge deck affects the aerodynamic characteristics of vehicles, thus affecting the driving safety of the bridge deck. Influenced by the mountain topography and the bridge deck's ancillary facilities, the local wind environment above the bridge deck is complex and changeable, and its impact on the bridge deck traffic can not be ignored. In order to accurately evaluate the local wind field parameters, a monitoring system of the local wind environment is developed. Utilizing the monitoring system, wind parameters above the approach deck of a long-span suspension bridge in a mountain area are measured. The relationship of wind characteristics between the incoming flow and the wind above the bridge deck is investigated. Results show the significant difference between the local wind environment above the bridge deck and the incoming flow's characteristics; the wind profile above the bridge deck does not follow the exponential distribution; the equivalent height of the wind load on the vehicle is higher than the vehicle's gravity centre. This study is relevant for studying the local wind environment, driving safety, and serviceability of long-span bridges in mountainous areas.

## Introduction

Investigation of the wind characteristics over the bridge deck has attracted significant attention in wind engineering. The recent construction trend of the long-span bridge in mountain areas has stimulated many studies on the local wind environment above the bridge deck. Since slender structures are susceptible to wind, wind load is the main determining factor in the safety and performance of these wind-sensitive structures. Similarly, vehicles are also sensitive to wind load^1,2^, and the local wind environment on the bridge deck has an important influence on the aerodynamic characteristics of vehicles^3^. Intense wind is one of the critical factors affecting vehicle safety on bridges, and its wind load creates hazards for structural integrity and driving safety^4,5^. The main beams of mountain long-span bridges are usually high from the ground to satisfy the requirements of line connectivity. As a result, the wind speed on the bridge deck is often high, which may lead to the deviation of the vehicle's route, side roll, and side slip of the vehicle, endangering driving comfort and even safety^6^.

In recent years, measured studies of wind characteristics in complex mountainous areas, such as a large number of field measurements of complex mountain areas in southwest China^7–10^, Norway^11,12^, etc., have confirmed that intense winds often invade the infrastructures in mountainous areas. In order to ensure the safe driving of vehicles on mountain bridges, attention should be paid to the characteristics of the local wind environment on the bridge deck. Finding out the wind parameter's distribution regularity of the wind environment on the bridge deck can not only optimize the ancillary facilities of the bridge deck, reduce the adverse impact on the wind environment caused by the ancillary facilities as much as possible, but also provide the necessary basis for vehicle driving safety.

The investigation of the local wind environment above the bridge deck is mainly carried out through means of wind tunnel test^12–14^, numerical simulation by CFD^15,16^, and field measurement. He et al. propose that the vehicle's driving position, such as its lateral and longitudinal bridge arrangement, significantly affects its aerodynamic coefficient, especially the lateral force coefficient^17^. The aerodynamic performance and dynamic response of the vehicle passing through the bridge tower under the lateral wind load are studied through CFD by Wang et al. Wang et al.^16^. Due to the complex and changeable flow field around the bridge tower, the vehicle is prone to side slip when passing through the bridge tower area. However, there is an apparent difference between the fluctuating wind and the incoming flow on the bridge deck, so it is not easy to consider the influence of the pulsating wind on the bridge deck by the numerical method. Methods of CFD and the wind tunnel test usually use uniform flow and ignore the pulsating components of the wind, despite the actual wind speed along the bridge may be non-uniform; moreover, it is difficult to accurately simulate the details of the bridge’s ancillary facilities in the model test, such as crash barrier, wind barrier, etc. A great deal of simplification of the actual wind and structure, the understanding of the wind characteristics of the bridge deck through CFD or wind tunnel test is limited, and the field measurement is the most effective method. The research on evaluating the local wind environment of the bridge deck by measuring wind characteristics and monitoring bridge response is mostly concentrated in plain and coastal regions^18,19^. Many studies^1,3,6,20^ have established evaluation models in terms of driving safety. However, these studies often aim at flat areas, which can not be directly applied to analyzing the local wind environment above the bridge deck in complex mountain areas. The variability of intense wind characteristics in mountain areas results in the complexity of the wind environment on the bridge deck.

Although the cost of field measurement is high and the number of measuring point is limited, the data of the local wind environment on the bridge deck can be directly obtained. Currently, the research on wind characteristics in mountain area is mainly around bridge sites, few investigations directly focus on the wind environment in the driving height on the bridge deck. For this reason, the focus of this paper is to analyze the local wind environment on the approach deck of a mountain long-span bridge through field measurement, paying attention to the relationship between the incoming flow and the wind above the bridge deck, as well as the mean and pulsation characteristics of the wind field over the bridge deck. The outline of the rest of the paper is as follows:

“Field measurement site, structure, and the measurement system” section introduces the topography of the measured mountain area, bridge structure, measurement system, and primary dataset. “Theory and methodology” section describes the methods of wind data processing and analysis. “Results and discussion” section discusses the distribution regularity of mean and fluctuating wind parameters and evaluates the equivalent wind speed of the vehicle according to the wind profile over the bridge deck. “Conclusions” section summarizes the main conclusions of this field measurement study.

## Field measurement site, structure, and the measurement system

### The topography and the bridge structure

**Figure 1:**
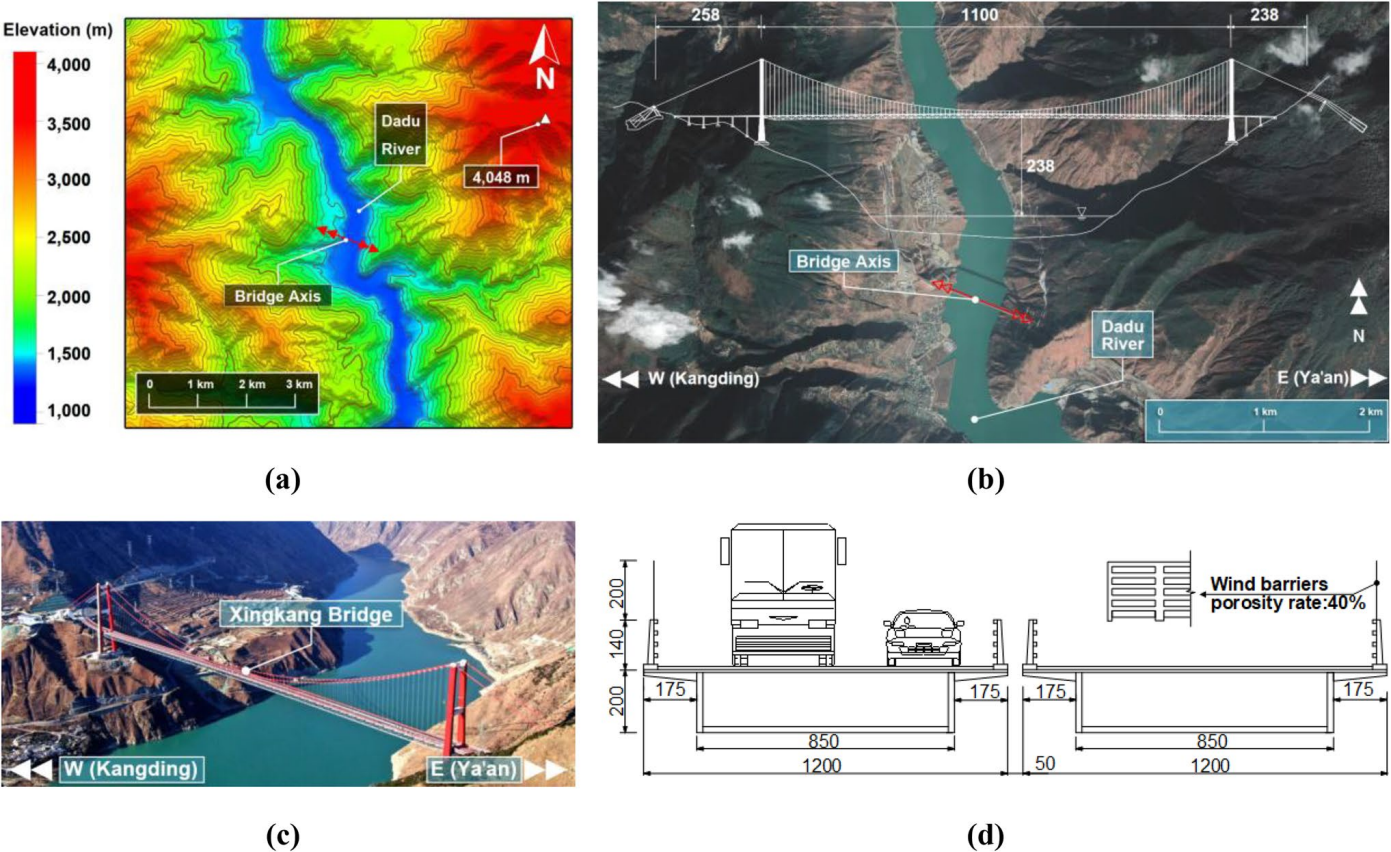
Topography and surroundings around the bridge site: (**a**) topographic contour map, (**b**) geomorphology map and overall layout of the Xingkang bridge (unit: m), (**c**) aerial view of Xingkang bridge, (**d**) cross section of approach bridge (unit: cm).

The field measurement was conducted at the Xingkang Bridge in the mountainous region of southwest China. The topographic contour and geomorphology map around the bridge are shown in Fig. 1a,b. High mountains and canyons surround this region with narrow terrain and ravines. The mountain peaks within 5 km range on both sides of the valley are more than 4000 m above sea level, covered with snow all year round, and are 3000 m higher than the bottom of the dry-hot valley. The mountains connected by the bridge are majestic, with a steep slope of 30°–50°, forming a typical U-shaped deep canyon. The bridge site is often affected by the southeast and southwest monsoon as well as the cold air over the Tibetan Plateau, causing the obvious difference in the vertical climate and leading to complex and changeable weather. Frequent disastrous weather can easily affect the driving safety on the bridge deck in this region, therefore, attention should be paid to the study of the local wind environment.

The main span of the bridge is an 1100-m-long steel truss beam. The west approach bridge is 6 × 34 m, and the east is 3 × 34 m. The bridge’s overall layout is shown in Fig. 1b, and the aerial view of the bridge is shown in Fig. 1c. Crossing the Dadu River, the suspension bridge’s main beam is 238 m higher than the river surface, ensuring road connectivity; but high elevation of the bridge deck causes the wind speed difference between the bridge deck and the canyon^21,22^, where the study of the local wind environment of the bridge deck is urgent. The approach bridge is a twin-box beam with a width of 12 m and a height of 2 m, and the 1.4 m-high crash barriers on both sides are each equipped with a 2 m-high strip wind barrier with 40% porosity rate (see Fig. 1d).

### Field monitor system

**Figure 2:**
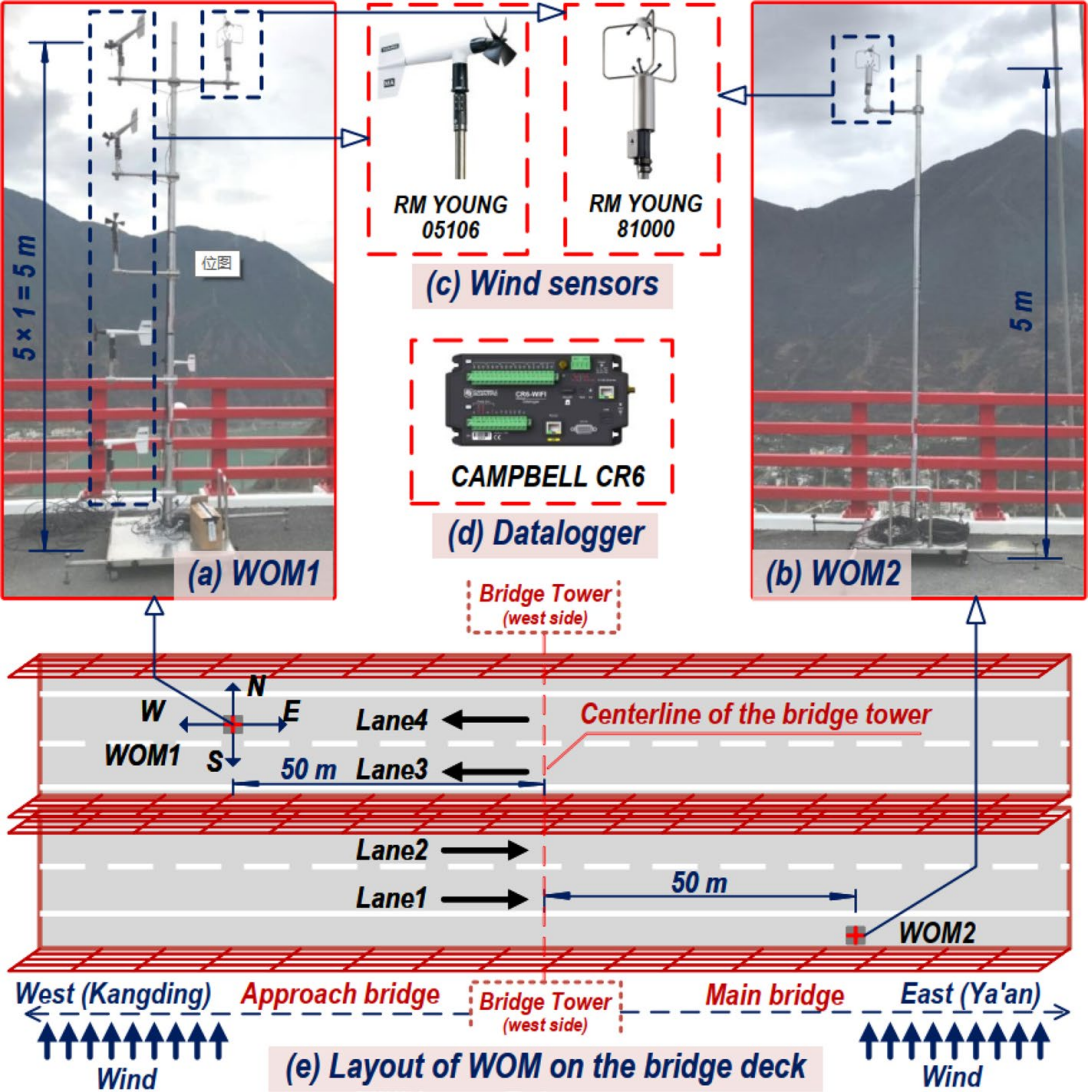
Monitoring system of wind environment on bridge deck: (**a**) WOM1, (**b**) WOM2, (**c**) wind sensors, (**d**) datalogger, (**e**) layout of WOM on the bridge deck.

Figure 2 exhibits the local wind environment monitor system on the bridge deck. Two 5 m-high movable wind parameter observation masts (hereinafter referred to as WOM) are developed to measure the wind speed above the bridge deck, which are divided into measuring masts (WOM1, see Fig. 2a) and reference masts (WOM2, see Fig. 2b). Each WOM comprises the wind measuring instruments, instrument brackets, main tower, and removable baseplate, and can be quickly arranged by manual or vehicle traction at any position on the bridge deck. The removable baseplate and main tower are made of steel. The baseplate’s cross-section is a square with a side length of 1.2 m, and the main tower is a circular steel tube with a diameter of 8 cm, ensuring the stability of the WOM. The wind measuring instruments on the two masts are arranged as follows:

- Measuring mast (WOM1, see Fig. 2a): An ultrasonic anemometer is installed at 5 m and five propeller anemometers at 1, 2, 3, 4, and 5 m above the ground. WOM1 is used to measure the wind speed time history on different lanes of the bridge deck;
- Reference mast (WOM2, see Fig. 2b): An ultrasonic anemometer is installed 5 m above the ground. WOM2 is fixed on the windward side of the bridge’s main span for long-term monitoring of wind speed time history.

The primary information about the two types of anemometers is shown in Table 1. The propeller anemometer is utilized to collect the wind speed profile data of the bridge deck, and the ultrasonic anemometer collects high-frequency wind data to analyze the mean and fluctuating wind parameters of the bridge deck's local wind environment. According to the author team's previous long-term wind speed measurement^22–24^, the wind direction at the bridge site is dominated by the south wind. Thus, the south side lanes (Lane 1 and Lane 2) are defined as the windward side lanes, and the north side lanes (Lane 3 and Lane 4) are the leeward side lanes. The arrangement of the masts on the bridge deck is shown in Fig. 2e. To ensure minimum interference of the measured wind data from bridge’s ancillary facilities as much as possible, the reference mast WOM2 is permanently fixed 50 m east of the west bridge tower and is close to the windward side crash barrier during the monitoring. It should be noted that there is no wind barrier on the main span’s crash barriers, so the data collected by WOM2 can represent the incoming wind data at the height of the bridge deck. The measuring tower WOM1 measures the wind data on the four lanes of the side-span approach bridge deck.

**Table 1:** List of the anemometer information.

Instrument type	Instrument model	Installed position (height from the bridge deck)	Recorded data	Frequency (Hz)	Range (m/s)	Accuracy
Ultrasonic anemometer	RM Young 81,000	WOM1: 5 m WOM2: 5 m	Instantaneous 3D wind speed	10	0–40	± 1% (0–30 m/s) ± 3% (30–40 m/s)
Propeller anemometer	RM Young 05,106	WOM1: 1, 2, 3, 4, 5 m	Wind speed and direction based on 10-min interval	1	0–100	± 1%

### Primary dataset

**Figure 3:**
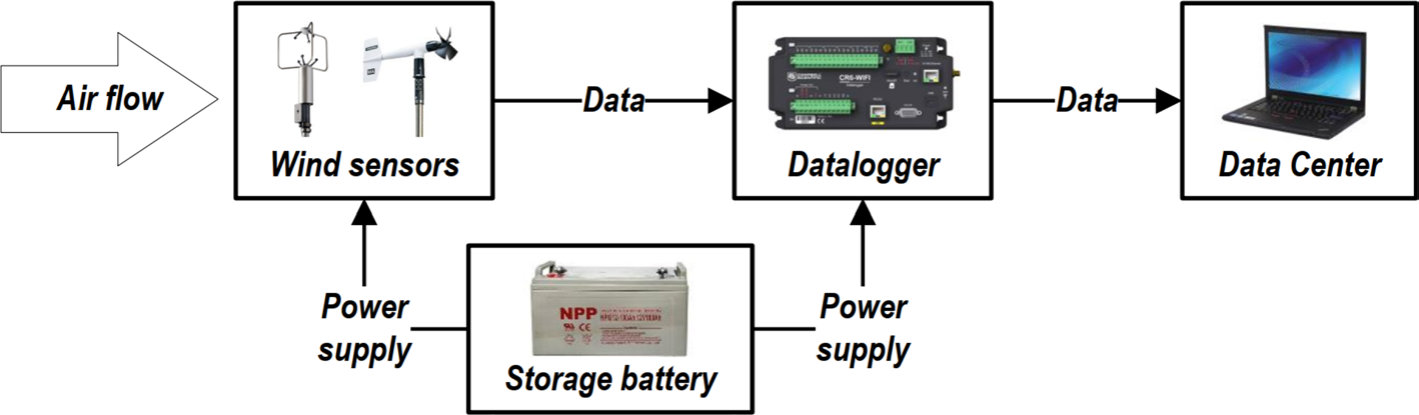
Flow chart of data record and transmission.

The datalogger’s model is CR6 (see Fig. 2d), which has 12 channels with a data acquisition rate of 125 Hz, meeting the requirement of the anemometer number and the requirement of high-frequency record of ultrasonic anemometers. Anemometers and collectors are powered by storage batteries. All instruments are calibrated before measurement, and WOM1 and WOM2 collect data synchronously during measurement. The monitor system first collects the incoming wind samples through the wind sensor, and the wind data is then collected by the datalogger and transmitted to the data center through a hard disk. The data record and transmission process is shown in Fig. 3. Follow-up analysis and discussion are carried out based on the wind data recorded from December 23, 2018, to December 30, 2018 (a total of 8 days).

## Theory and methodology

This section introduces the processing of wind data and the calculation method of wind parameters, including the mean wind parameters, fluctuating wind parameters, wind profile, and equivalent wind speed for driving safety. The mean and fluctuating wind field parameters such as mean wind speed, wind direction, gust factor, turbulence intensity, and power spectrum are calculated from the wind data recorded by 5 m-high ultrasonic anemometers. The wind profile and equivalent wind speed for driving safety are computed from the wind data recorded by five propeller anemometers with gradient arrangement.

### Mean wind parameters

Mean wind speed and mean wind direction are important factors affecting the operating environment of vehicles on the bridge. At an interval of 10 min, the 10-min mean wind speed $\overline{U }$ and mean wind direction $\phi$ can be obtained from the wind speed time history along the north ${(U}_{x}\left(t\right))$, east ${(U}_{y}\left(t\right))$ and vertical ${(U}_{z}\left(t\right))$ direction collected by the ultrasonic anemometer as follows:

1 $$\overline{U }=\sqrt{{\overline{U} }_{x}^{2}+{\overline{U} }_{y}^{2}+{\overline{U} }_{z}^{2}}$$

2 $$\varphi =\mathrm{arccos}\left(\frac{{\overline{U} }_{x}}{\sqrt{{\overline{U} }_{x}^{2}+{\overline{U} }_{y}^{2}}}\right)$$

where ${\overline{U} }_{i}$ are the 10-min mean value of ${U}_{i}\left(t\right) (i=x, y,z)$; the mean wind direction $\phi$ is calculated in degrees (°), 0° represents the north direction, and 90° represents the east.

### Fluctuating wind field parameters

After obtaining the mean wind speed $\overline{U }$ by Eq. (1), the fluctuating speed in three directions of the wind axis can be expressed by Eq. (3), that is, the longitudinal component $u\left(t\right)$, lateral component $v\left(t\right)$, vertical component $w\left(t\right)$. Then the gust factor ${G}_{i}$ and turbulence intensity ${I}_{i}$ can be calculated from Eqs. (4), (5), which characterize the degree of turbulence at the atmospheric boundary.

3 $$\left\{\begin{array}{l}U\left(t\right)=\overline{U }+u\left(t\right)\\ V\left(t\right)=v\left(t\right)\\ W\left(t\right)=w\left(t\right)\end{array}\right.$$

4 $$\left\{\begin{array}{l}{G}_{u}=1+\frac{{u}_{\mathrm{max},\uptau }}{\overline{U}}\\ {G }_{i}=\frac{{i}_{\mathrm{max},\uptau }}{\overline{U} },(i=v, w)\end{array}\right.$$

5 $${I}_{i}=\frac{{\sigma }_{i}}{\overline{U} } \left(i=u,v,w\right)$$

where ${i}_{\mathrm{max},\uptau } (i=u,v,w)$ are the maximum fluctuating components in three directions within the gust duration $\uptau$, which is 3 s in this study^25,26^; ${\sigma }_{i} (i=x, y,z)$ are the root mean squares of the fluctuating components.

The power spectrum of fluctuating wind expresses the contribution of turbulent kinetic energy in various frequencies. Several classical power spectral density functions (PSD) can estimate the measured power spectrum. Chinese wind-resistant design specification for highway bridges^27^ recommends Simiu spectrum^28^ for longitudinal spectrum, Kaimal spectrum^29^ for lateral spectrum, and Panofsky spectrum^30^ for vertical spectrum. These three forms of PSD are shown in Eq. (6):

6 $$\frac{f\cdot {S}_{i}\left(f,z\right)}{{u}_{*}^{2}}=\frac{{A}_{i}{f}_{z}}{{\left(1+{B}_{i}{f}_{z}\right)}^{{\alpha }_{i}}} \left(i=u,v,w\right)$$

where ${S}_{i}\left(f,z\right) \left(i=u,v,w\right)$ is the longitudinal, lateral, and vertical power spectrum at height z; $f$ is the frequency; ${u}_{*}$ is the friction velocity of the airflow; ${f}_{z}$ is the Morning similar coordinate (reduced frequency) where ${f}_{z}=f\cdot z/\overline{U }$; ${A}_{i}, {B}_{i},{\alpha }_{i}(i=u,v,w)$ are the dimensionless coefficients corresponding to three directions: for the longitudinal spectrum, Simiu and Scanlan recommend ${A}_{u}=200,{B}_{u}=50,{\alpha }_{u}=5/3$^28^; for the transverse spectrum, Kaimal et al. recommends ${A}_{v}=17,{B}_{v}=9.5,{\alpha }_{v}=5/3$^29^; Panofsky and McCormick^30^ recommend ${A}_{w}=6,{B}_{w}=4,{\alpha }_{w}=2$. The above classical PSDs can well describe the measured wind power spectra in coastal, plain or other open areas, but in recent years, the measured results of wind fields in many mountainous areas show that these classical spectra may misestimate the mountain measured turbulent kinetic energy, while the von Kármán spectrum^31^ (see Eq. (7)) is in better agreement with the measured results^9,22,32,33^. However, the analysis of the von Kármán spectrum is more complicated, involving the calculation of the integral turbulence scale ${L}_{i}$ that requires simultaneous measurement of multiple points in space, so it is difficult to achieve in engineering and can only be estimated by approximate methods. The estimated results in different topography or approximate methods have great variability^11,34,35^. Hence, this study takes the form of Simiu, Kaimal, and Panofsky’s PSD. According to the spectrum normalization method of von Kármán’s PSD, the power spectra in three directions are described by Eq. (8), the fitting method is used to determine the dimensionless coefficient ${A}_{i}, {B}_{i},{\alpha }_{i}(i=u,v,w)$. In particular, the power spectrum is a function of ${f}_{z}^{-{\alpha }_{i}}$^36^, so the power exponent coefficient ${\alpha }_{i}$ is determined by independent fitting.

7 $$\frac{f\cdot {S}_{i}(f,z)}{{\sigma }_{i}^{2}}=\frac{4\times \frac{{L}_{i}f}{U}\times \left[1+{A}_{i}\times {\left(\frac{{L}_{i}f}{\overline{U} }\right)}^{2}\right]}{{\left(1+{B}_{i}\times {\left(\frac{{L}_{i}f}{\overline{U} }\right)}^{2}\right)}^{{\alpha }_{i}}} \left(i=u,v,w\right)$$

8 $$\frac{f\cdot {S}_{i}\left(f,z\right)}{{\sigma }_{i}^{2}}=\frac{{A}_{i}{f}_{z}}{{\left(1+{B}_{i}{f}_{z}\right)}^{{\alpha }_{i}}} \left(i=u,v,w\right)$$

### Wind profile and equivalent wind speed for driving safety

Wind speed profile is significant for bridge deck driving safety and comfort. In wind engineering, it is often assumed that wind profile accords with exponential distribution^37–39^; but according to the field measured data in mountain terrain^40^, the mountain wind profile is closer to the S-shaped curve. Therefore, the quartic polynomial function is used to fit the wind profile as follows:

9 $${U}_{z}/{U}_{0}={p}_{1}{z}^{4}+{p}_{2}{z}^{3}+{p}_{3}{z}^{2}+{p}_{4}z+{p}_{5}$$

where ${U}_{z}$ is the wind speed at height $z$ above the bridge deck collected by the propeller anemometers on WOM1; ${U}_{0}$ is the incoming flow speed collected by WOM2 during the same period. The wind profiles at different lanes are observed from WOM1 sequentially but not simultaneously. To compare the changes of the wind profile above different lanes, the wind profile measured by the propeller anemometers, hence, will be normalized based on the wind speed of the reference mast WOM2; ${p}_{i} (i=1, 2, 3, 4)$ are parameters to be fitted, particularly when $z=0$, the speed at the surface of the bridge deck should be 0, so ${p}_{5}=0$.

The equivalent wind speed ${V}_{e}$ represents the bridge deck wind environment on driving safety and the wind speed reduction coefficient ${\lambda }_{r}$ evaluates the influence of bridge deck ancillary facilities on the incoming wind. Based on the principle of equivalent lateral force and equivalent overturning moment, the corresponding equivalent wind speed ${V}_{eF}$ and ${V}_{eM}$ can be calculated through Eqs. (10), (11) ^41,42^. Then, the wind speed reduction coefficient ${\lambda }_{r}$ can obtained by Eq. (12). The higher the ${\lambda }_{r}$, the greater the interference of the bridge deck ancillary facilities to the incoming wind speed.

10 $${V}_{eF}=\sqrt{\frac{1}{{Z}_{e}}{\int }_{0}^{{Z}_{e}}{U}^{2}\left(z\right)dz}$$

11 $${V}_{eM}=\sqrt{\frac{1}{{Z}_{e}^{2}}{\int }_{0}^{{Z}_{e}}z\cdot {U}^{2}\left(z\right)dz}$$

12 $${\lambda }_{r}=\frac{{U}_{0}-{V}_{e}}{{U}_{0}}$$

where ${Z}_{e}$ is the equivalent height.

## Results and discussion

### Mean wind speed and wind direction

**Figure 4:**
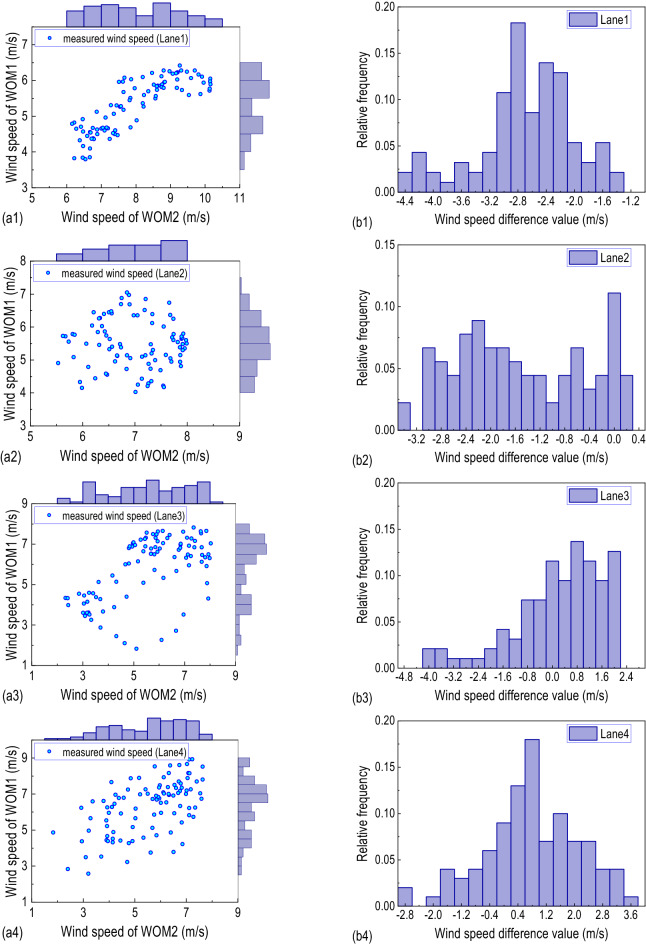
Probability distribution of wind speed at different lanes: (**a**) wind speed distribution of two masts; (**b**) wind speed difference probability distribution between two masts; (1) Lane 1, (2) Lane 2, (3) Lane 3, (4), Lane 4.

This part mainly discusses the difference in mean wind speed and wind direction between the incoming flow and the wind samples on the bridge deck. Figure 4a gives the distribution relationship between the wind speed at different lanes collected by WOM1 and the incoming wind speed collected by WOM2. Figure 4b shows the wind speed difference between WOM1 and WOM2. From the probability distribution of the wind speed measured by the two masts, due to the shielding effect of the bridge tower and the wind barrier^43^, there is a great discreteness in wind speed and little correlation between the measuring mast WOM1 and the reference mast WOM2. The difference in wind speed between the two shows that the windward wind barrier effectively reduces the wind speed above the upwind side lanes (Lane 1 and Lane 2). The incoming wind speed decreases by about 3 m/s above Lane 1, while the wind speed above the leeward side lines (Lane 3 and Lane 4) becomes greater than the incoming wind speed.

**Figure 5:**
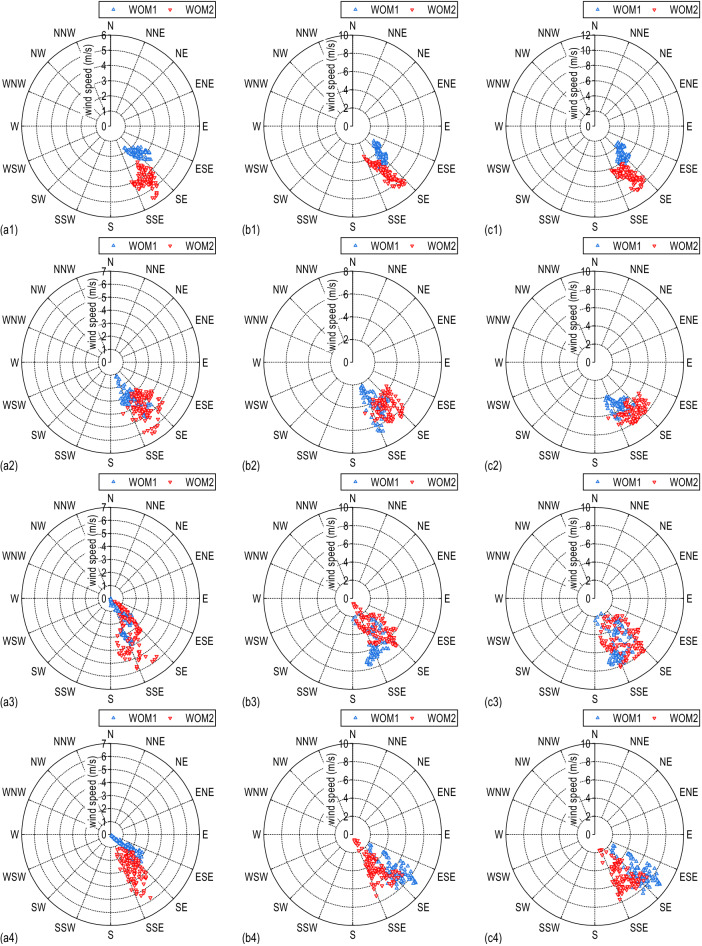
Wind rose of wind direction and: (**a**) lateral bridge wind speed, (**b**) longitudinal bridge wind speed, (**c**) total wind speed; (1) Lane 1, (2) Lane 2, (3) Lane 3, (4) Lane 4.

Figure 5c gives the wind speed rose diagram of the total wind speed. The dominant wind direction of the incoming wind is the southeast direction. The direction differs between the incoming flow and the wind above the lanes. As mentioned in Fig. 4b1, the total wind speed above Lane 1 is much lower than the incoming flow. During the measured period, the mean value of wind speed above lane 1 is 5.3 m/s, while the corresponding value of incoming wind is about 8.0 m/s. As the measuring mast WOM1 moves laterally to leeward side lanes, the wind speed and direction difference between WOM1 and WOM2 gradually decreases, and the wind speed above Lane 4 is even higher than the incoming wind speed. Decompose the wind speed into the lateral bridge wind speed and the longitudinal bridge wind speed, shown in Fig. 5a,b. After decomposing the wind speed, the wind speed distribution of the lateral bridge is different from that of the longitudinal bridge, and the mean lateral bridge wind speed measured by the measuring mast WOM1 on the four lanes is lower than that of the reference mast WOM2. The mean value of the lateral bridge wind speed on WOM1 above Lane 1 ~ Lane 4 is 2.53 m/s, 3.20 m/s, 1.98 m/s and 1.78 m/s, respectively, the corresponding speed on reference mast WOM2 is 4.22 m/s, 4.26 m/s, 3.06 m/s and 3.07 m/s. The normalized lateral bridge wind speed based on the WON2 from Lane 1 to Lane 4 is 0.599, 0.751, 0.650, and 0.580, respectively. Therefore, the wind barrier effectively reduces the lateral bridge wind speed and significantly improves the local mean wind field on the bridge deck. For the longitudinal bridge wind speed, the longitudinal bridge wind speed on the windward side lanes (Lane 1 and Lane 2) is lower than that of the incoming longitudinal bridge, due to the shielding effect of the windward side wind barrier. As the WOM1 moves to the leeward side lanes (Lane 3 and Lane 4), the shielding effect of the windward wind barrier gradually weakens; meanwhile, the wind barrier has a guiding effect on the local longitudinal wind field of the bridge deck; therefore, the longitudinal bridge wind speed at the leeward side is higher than the incoming flow.

### Gust factor

**Figure 6:**
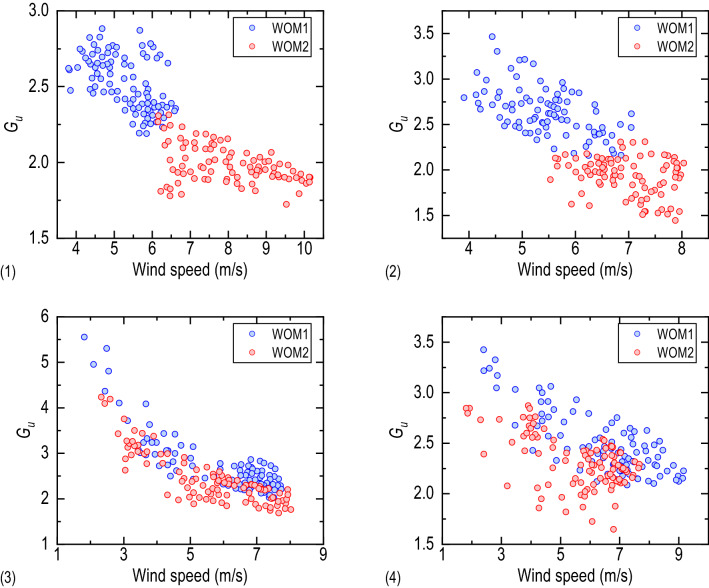
The Relationship between gust factor and wind speed at: (1) Lane 1, (2) Lane 2, (3) Lane 3, (4) Lane 4.

Gust factor can be used to measure the pulsation characteristics of airflow and has a noticeable impact on vehicles. The variation regularity of the lateral bridge gust factor with wind speed in different lanes is shown in Fig. 6. For both the reference mast and the measuring mast, the gust factor decreases gradually with the increase of wind speed, but exhibits an apparent difference in the value of the gust factor between the measuring mast and the reference mast. Directly affected by the wind barrier, the gust factor above the windward side lanes is much larger than that of the incoming wind. For the leeward side lanes, the difference in the gust factor between the two masts weakens, but the gust factor above the lanes is still higher than the incoming flow. Table 2 gives the mean value of the gust factor at different lanes. The lateral bridge gust factor above the lanes is higher than that of the incoming wind, and the increase in the gust factor of the lanes on the windward side is much higher than that on the leeward side. For the same lane, the longitudinal bridge and vertical gust factor above the lanes are lower than that of the incoming wind, and the decrease on the leeward side is higher than that on the windward side.

**Table 2:** Mean value of gust factor at different lanes.

		Lane 1	Lane 2	Lane 3	Lane 4
*G*_*u*_	WOM1	2.53	2.64	2.76	2.48
	WOM2	1.99	1.93	2.45	2.32
*G*_*v*_	WOM1	1.10	1.05	1.25	1.11
	WOM2	1.12	1.36	1.64	1.56
*G*_*w*_	WOM1	0.36	0.43	0.43	0.32
	WOM2	0.60	0.70	0.92	0.91

### Turbulence intensity

**Figure 7:**
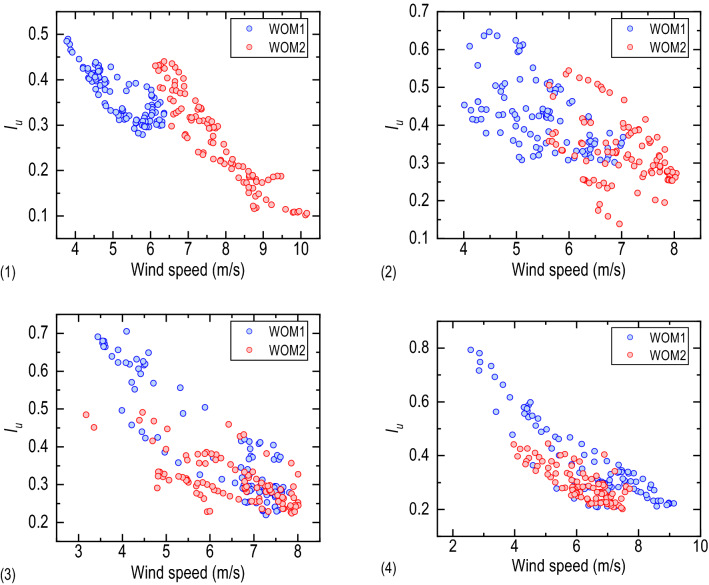
The relationship between turbulence intensity and wind speed at: (1) Lane 1, (2) Lane 2, (3) Lane 3, (4) Lane 4.

Turbulence intensity is a parameter for judging the fluctuation of the wind field as well as vehicle driving safety and comfort. Figure 7 shows the variation of the lateral bridge turbulence intensity with the wind speed at different lanes. With the increase of wind speed, turbulence intensity and its discreteness decrease gradually. Under the direct influence of the wind barrier, the turbulence intensity above Lane 1 is higher than the incoming wind, and there is a clear separation between the turbulence intensity of WOM1 and WOM2. The turbulence intensity from Lane 2 to Lane 4 is also higher than that of the incoming wind, but there is a particular coincidence between the turbulence intensity of WOM1 and WOM2. Table 3 gives the mean value of turbulence intensity at different lanes. The mean turbulence intensity in the three directions above each lane is higher than that of the incoming wind. The turbulence intensity ratio ${I}_{u}:{ I}_{v}: {I}_{w}$ from Lane 1 to Lane 4 is 1:1.21:0.73, 1:1.06:0.64, 1:1.05:0.58, 1:1.13:0.53, respectively, differing from the recommended value of 1:0.88:0.50 by Chinese wind-resistant design specification^27^; thus, the recommended value can not be directly applied to describe the local wind environment of the bridge deck.

**Table 3:** Mean value of turbulence intensity at different lanes.

		Lane 1	Lane 2	Lane 3	Lane 4
*I*_*u*_	WOM1	0.363	0.425	0.395	0.382
	WOM2	0.264	0.327	0.313	0.292
*I*_*v*_	WOM1	0.442	0.452	0.411	0.431
	WOM2	0.287	0.308	0.275	0.251
*I*_*w*_	WOM1	0.264	0.274	0.228	0.204
	WOM2	0.190	0.221	0.183	0.173

### Power spectrum

**Figure 8:**
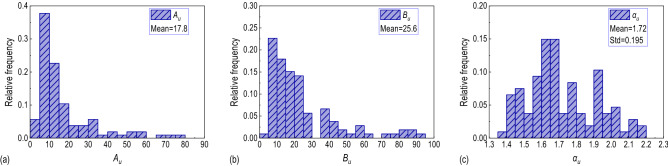
The frequency distribution histogram of the fitting parameters: (**a**) ${A}_{u}$, (**b**) ${B}_{u}$, (**c**) ${\alpha }_{u}$.

The measured power spectra in three directions are fitted according to Eq. (8). Figure 8 shows the frequency distribution of the dimensionless fitting coefficients in the longitudinal direction. The fitting coefficients in the other two directions are similar. The mean value of ${\alpha }_{i}(i=u,v,w)$ is 1.72,1.53 and 1.48 respectively. Comparing to the recommended values of the Simiu spectrum, Kaimal spectrum, and Panofsky spectrum mentioned in Eq. (6), which are 5/3, 5/3, and 2, respectively, ${\alpha }_{u}$ and ${\alpha }_{v}$ are relatively close to 5/3, while ${\alpha }_{w}$ is much less than 2, indicating that the fluctuating wind in the horizontal plane of the bridge deck is significantly affected by the incoming wind, and the vertical fluctuating wind is influenced by the ancillary structure of the bridge deck. In the same way, the mean value of ${A}_{i}$ and ${B}_{i}\left(i=u,v,w\right)$ are ${A}_{u}=17.8, {B}_{u}=25.6$; ${A}_{v}=22.3, {B}_{v}=46.8$; ${A}_{w}=7.4, {B}_{w}=12.5$. The fitting power spectrum functions in three directions of the bridge deck can be expressed in Eqs. (13)–(15).

**Figure 9:**
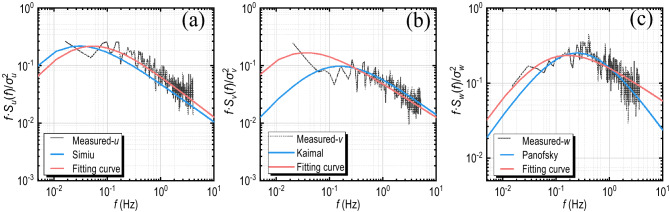
The power spectrum of a measured sample over the bridge deck: (**a**) *u*; (**b**) *v*; (**c**) *w.*

Figure 9 compares the measured power spectrum, the fitting spectrum, and some classical spectra of a wind sample on the bridge deck. The variation regularity of the fitting spectrum is consistent with that of the measured samples. The selected ${\alpha }_{i}$ values reflect the changing trend of the measured power spectrum well, and the fitted wind spectrum describes the turbulent kinetic energy at high frequency or low frequency well. For the classical spectrum recommended by the specification, especially the Simiu spectrum and Panofsky spectrum, although the trend of frequency variation is similar to that of the measured spectrum, there is an obvious deviation between the energy and the measured result.

13 $$\frac{f\cdot {S}_{u}\left(f,z\right)}{{\sigma }_{u}^{2}}=\frac{17.8{f}_{z}}{{\left(1+25.6{f}_{z}\right)}^{1.72}}$$

14 $$\frac{f\cdot {S}_{v}\left(f,z\right)}{{\sigma }_{v}^{2}}=\frac{22.3{f}_{z}}{{\left(1+46.8{f}_{z}\right)}^{1.59}}$$

15 $$\frac{f\cdot {S}_{w}\left(f,z\right)}{{\sigma }_{w}^{2}}=\frac{7.4{f}_{z}}{{\left(1+12.5{f}_{z}\right)}^{1.48}}$$

### Wind profile and evaluation of equivalent wind speed

**Figure 10:**
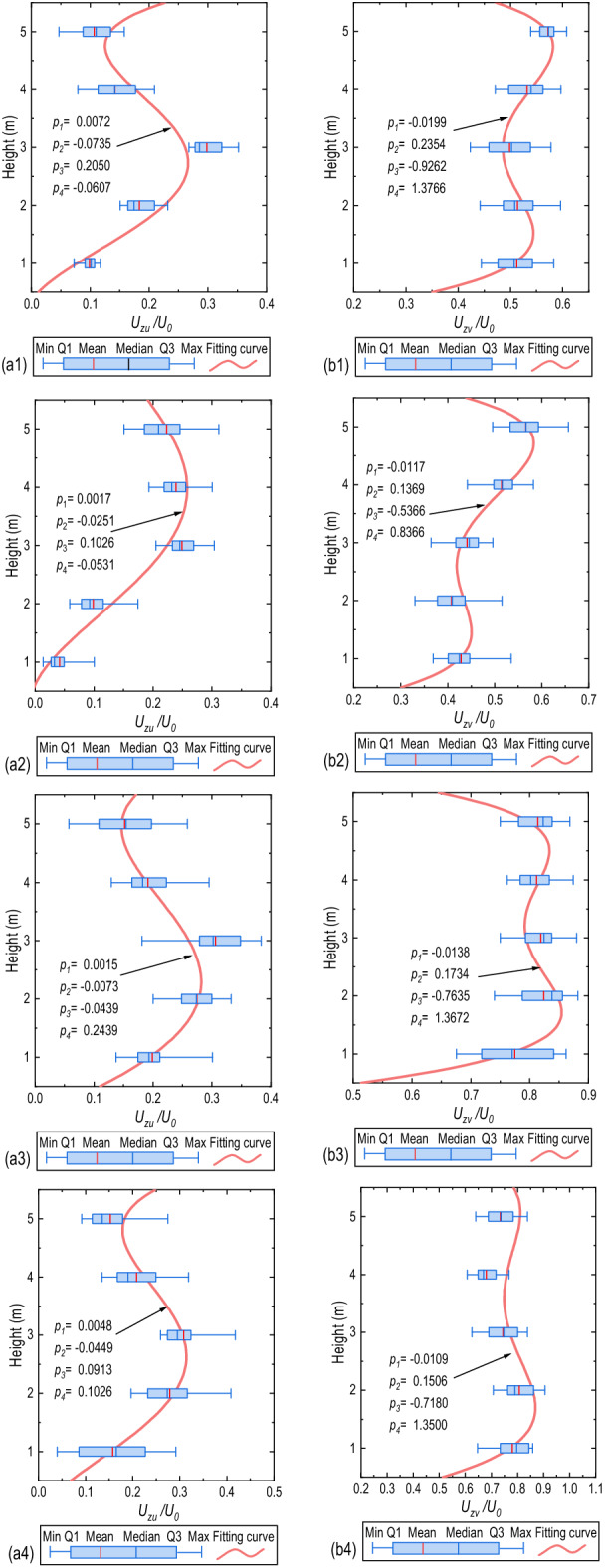
Boxplot of measured wind profile and fitting curve above different lanes: (**a**) lateral wind profile, (**b**) longitudinal wind profile; (1) Lane 1, (2) Lane 2, (3) Lane 3, (4) Lane 4.

Figure 10 shows the variation regularity of the normalized wind profile in the lateral and longitudinal directions. The wind speed’s variation trends with the height in two directions differ. In the range of measured height, with increasing height, the normalized wind speed along the lateral bridge direction first increases and then decreases slightly, while the normalized wind speed along the longitudinal bridge direction decreases at first and then increases. As the WOM1 moves to the leeward lanes, the difference in wind speed within different heights weakens. The fitted results according to Eq. (9) are also shown in Fig. 10. The quartic polynomial function better describes the wind profile in different lanes and directions in the range of measured height. The shape of the fitting curve is quite different between the lateral bridge wind profile and the longitudinal bridge wind profile.

The equivalent wind speeds of the travelling bus and articulated lorry are evaluated in this research. Their body heights are 3.3 m and 3.78 m, respectively, and the height of the gravity centre is 1.4 m and 1.7 m^14,44^. According to the fitted function of the lateral bridge wind profile, the equivalent wind speed in the height range of the two vehicles is calculated, and the equivalent height of the equivalent wind speed is calculated based on the torque equivalence. The normalized equivalent wind speed ${V}_{e}$, the equivalent height ${Z}_{e}$, and wind speed reduction coefficient ${\lambda }_{r}$ calculated through Eqs. (10)–(12) are shown in Table 4. From Lane 1 to Lane 4, the equivalent wind speed decreases gradually. The local wind speed on the leeward side of the bridge deck is conducive to the safety of vehicles. However, although the wind barrier dramatically reduces the equivalent wind speed acting on the vehicle, the equivalent height of the wind load is still higher than the actual gravity centre of the vehicle. Therefore, attention should be paid to driving comfort and safety, such as height limit in traffic during intense winds.

**Table 4:** Normalized equivalent wind speed based on fitting wind profile in lateral direction.

Number	Vehicle type	${V}_{e}$		${\lambda }_{r}$		${Z}_{e}$ (m)
		${V}_{eF}$	${V}_{eM}$	${\lambda }_{rF}$	${\lambda }_{rM}$	
Lane 1	Coach	0.1849	0.1580	0.8151	0.8420	2.33
	Towed truck	0.1902	0.1580	0.8098	0.8420	2.54
Lane 2	Coach	0.1314	0.1176	0.8686	0.8824	2.47
	Towed truck	0.1518	0.1343	0.8482	0.8657	2.85
Lane 3	Coach	0.2276	0.1809	0.7724	0.8191	2.03
	Towed truck	0.2272	0.1759	0.7728	0.8241	2.22
Lane 4	Coach	0.2352	0.1950	0.7648	0.8050	2.22
	Towed truck	0.2397	0.1938	0.7603	0.8062	2.43

## Conclusions

Based on the developed local wind environment monitor system, the wind field measurement study on the approach bridge of a long-span suspension bridge in a mountain area is conducted, and the wind parameters at different lanes of the approach bridge deck are tested. The following conclusions can be drawn:

1. The southeast wind dominates the wind direction above the bridge deck. The mean wind characteristics above the bridge deck are influenced by the ancillary facilities such as bridge towers and wind barriers. The wind barrier reduces the lateral bridge wind speed and has a guide effect on the longitudinal bridge wind speed, increasing it slightly.
2. The fluctuating wind characteristics above the bridge deck are influenced by the ancillary facilities. The wind barrier enhances the gust factor and turbulence intensity above the bridge deck. The growth range of the lateral bridge gust factor gradually becomes smaller as the lane moves to the leeward side, and the mean turbulence intensity along the lateral bridge is higher than that along the longitudinal bridge.
3. The fitting power spectrum above the bridge deck determined by the fitted mean value better exhibits the energy distribution and energy variation trend with the frequency of the measured power spectrum over the bridge deck.
4. The exponential distribution law around the bridge site is insufficient in describing the wind profile above the bridge deck. The Wind barrier reduces the equivalent wind speed based on lateral force and overturning moment, but the equivalent action height of wind load on the two kinds of vehicles is higher than the height of the gravity centre, which is not conducive to driving vehicles.

After the local wind environment parameters of the bridge deck were measured, it is necessary to evaluate the safety and comfort of the bridge deck vehicle driving in the mountainous area through the wind-vehicle-bridge coupling analysis. The local wind environment monitoring system for the bridge deck developed in this paper is also suitable for bridges in different regions. The research results could serve as a reference for the wind-resistant design of the bridge, provide a basis for the study of the wind environment on the bridge deck, and provide suggestions for vehicle driving safety and driving comfort in complex mountainous areas.

## Data Availability

The datasets generated during and/or analysed during the current study are available from the corresponding author on reasonable request.
